# Rice Bran Feruloylated Oligosaccharides Activate Dendritic Cells via Toll-Like Receptor 2 and 4 Signaling

**DOI:** 10.3390/molecules19045325

**Published:** 2014-04-23

**Authors:** Chi Chen Lin, Hua Han Chen, Yu Kuo Chen, Hung Chia Chang, Ping Yi Lin, I-Hong Pan, Der-Yuan Chen, Chuan Mu Chen, Su Yi Lin

**Affiliations:** 1 Institute of Biomedical Science, National Chung-Hsing University, Taichung 402, Taiwan; 2 Department of Medical Research and Education, Taichung Veterans General Hospital, Taichung, 407, Taiwan; E-Mail: dychen@mail.vghtc.gov.tw; 3 Rong Hsing Research Center for Translational Medicine, National Chung Hsing University, Taichung 402, Taiwan; E-Mail: chchen1@dragon.nchu.edu.tw; 4 Division of Chest Medicine, Department of Internal Medicine, Changhua Christian Hospital, Changhua 500, Taiwan; 5 Department of Food Science, National Penghu University of Science and Technology, Makung City, Penghu Hsien 880, Taiwan; E-Mail: demonsandy@gmail.com; 6 Department of Food Science, National Pingtung University of Science and Technology, Pingtung 912, Taiwan; E-Mail: chenyk@mail.npust.edu.tw; 7 Transplant Medicine & Surgery Research Centre, Changhua Christian Hospital, Changhua 500, Taiwan; E-Mail: lillian1109ms@yahoo.com.tw; 8 Biomedical Technology and Device Research Laboratories, Industrial Technology Research Institute, Hsinchu 310, Taiwan; E-Mail: I-HorngPan@itri.org.tw; 9 Department of Life Sciences, National Chung Hsing University, Taichung 402, Taiwan; 10 Department of Applied Science of Living, Chinese Culture University, Taipei 111, Taiwan; E-Mail: ajeff1002@hotmail.com

**Keywords:** dendritic cells, feruloylated oligosaccharides, maturation, rice bran, Toll-like receptor (TLR)

## Abstract

This work presents the effects of feruloylated oligosaccharides (FOs) of rice bran on murine bone marrow-derived dendritic cells (BMDCs) and the potential pathway through which the effects are mediated. We found that FOs induced phenotypic maturation of DCs, as shown by the increased expression of CD40, CD80/CD86 and MHC-I/II molecules. FOs efficiently induced maturation of DCs generated from C3H/HeN or C57BL/6 mice with normal toll-like receptor 4 (TLR-4) or TLR-2 but not DCs from mice with mutated TLR4 or TLR2. The mechanism of action of FOs may be mediated by increased phosphorylation of ERK, p38 and JNK mitogen-activated protein kinase (MAPKs) and increased NF-κB activity, which are important signaling molecules downstream of TLR-4 and TLR-2. These data suggest that FOs induce DCs maturation through TLR-4 and/or TLR-2 and that FOs might have potential efficacy against tumor or virus infection or represent a candidate-adjuvant approach for application in immunotherapy and vaccination.

## 1. Introduction

Ferulic acid (4-hydroxy-3-methoxycinnamic acid; FA) is one of the most abundant, ubiquitous hydroxycinnamic acids derived from phytochemical phenolic compounds. In cereals, significant levels of FA are in an insoluble bound form, esterified with the arabinose of arabinoxylans in the cell wall [1]. Treatment of Gramineae tissues with partial enzymatic or mild acid hydrolysis yielded FOs, which were purified with the subsequent chromatographic fractionation. FOs involve various oligosaccharides ester-linked to ferulic acid, such as feruloylated disaccharide O-[5-O-(trans-feruloyl)-α-l-Ara f]-(1→3)-d-Xyl p, and the feruloylated trisaccharide derived from arabinoxylans, O-[5-O-(feruloyl)-α-l-Ara f]-(1→3)-O-β-d-Xyl p-(1→4)a-d-Xyl p [2,3]. FOs have shown many functional properties, such as their antioxidant characteristics and immunostimulatory activity in macrophages, which was proved by increasing the production of TNF-α, IL-1β, IL-6, NO and PGE2 in RAW 264.7 [3,4]. These functions suggested that FOs might have potential for use in atherosclerosis prevention or in other applications where their antioxidant and immunostimulatory capacities would be a valuable asset, such as in the health food, pharmaceutical and cosmetic industries.

Dendritic cells (DCs) are professional antigen-presenting cells (APCs) that play the unique role of immune sentinels by initiating T cells responses and linking innate and adaptive immunity [5,6]. DCs are present at different stages of maturation in the circulation and in nonlymphoid and lymphoid organs. DCs reside in an immature form in peripheral nonlymphoid tissues, where they capture and process exogenous antigens [7]. Thereafter, DCs migrate to the T cell areas of the lymphoid organs, where they present antigenic peptides to T lymphocytes and stimulate naïve T cell responses through cytokine signals, major histocompatibility complex (MHC) molecules and co-stimulatory molecules (e.g., CD40, CD80 and CD86) [8]. As key regulatory mediators of immune responses, DCs are being pursued for the development of potent new vaccines against cancer and infectious disease [9]. In addition, the identification of materials that can modulate DCs activation and function is an emerging field that can develop alongside DCs immunobiology [10]. Therefore, natural or synthetic activators that promote DCs activation may potentially be candidate adjuvants for application in immunotherapy and vaccination.

In this research, we aim to investigate the effect of FOs of rice bran on immune response and its potential cellular targets. Herein, we evaluate whether FOs affect the maturation and functional properties of murine bone marrow-derived dendritic cells (BMDCs).

## 2. Results

### 2.1. FOs Increase Cytokine and Chemokine Production by BMDCs

**Figure 1:**
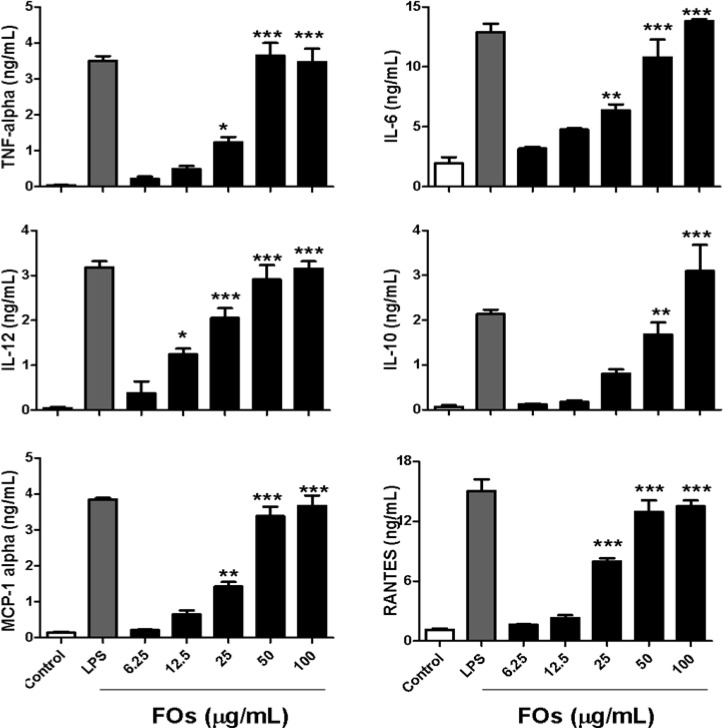
Cytokine and chemokine production are increased in BMDCs stimulated with FOs (12.5–100 g/mL) for 24 h (6 h for the TNF-alpha). The data are presented as the mean ± SD from triplicate well measurements from one of three independent experiments with similar results. * *p* < 0.05, ** *p* < 0.01, *** *p* < 0.001 was for the comparison between FOs-treated and PBS-treated control DCs.

Mature DCs are characterized by the synthesis and secretion of pro-inflammatory cytokines, chemokines, and up-regulation of surface co-stimulatory molecules and major histocompatibility complex molecules [5,6,7]. Therefore, in the first series of experiments, we investigated the effect of FOs on the secretion of the selective pro-inflammatory cytokines TNF-alpha, IL-6, IL-10 and the Th1 cytokine IL-12 in the supernatants of BMDCs by sandwich ELISA. BMDCs treated with LPS were used as a positive control and PBS as a negative control. The results in Figure 1 indicate that DCs treated with FOs dosages of 25 μg/mL or more, significantly increase TNF-alpha, IL-6, IL-10 and IL-12 production as compared to the control group, in a dose-dependent manner. Similar to FOs, LPS significantly enhanced these cytokines compared to the control group. The maximal cytokine levels induced by LPS (100 ng/mL) are comparable to the maximal levels induced by 50 μg/mL FOs. There is a similar tendency in the production of chemokines induced by FOs. Figure 1 shows that FOs (25–100 μg/mL) stimulated DCs to produce substantial amounts of dose-dependent MCP-1, and RANTES.

### 2.2. FOs Up-Regulate the Expression of Immunomodulatory Cell Surface Markers on BMDCs

**Figure 2:**
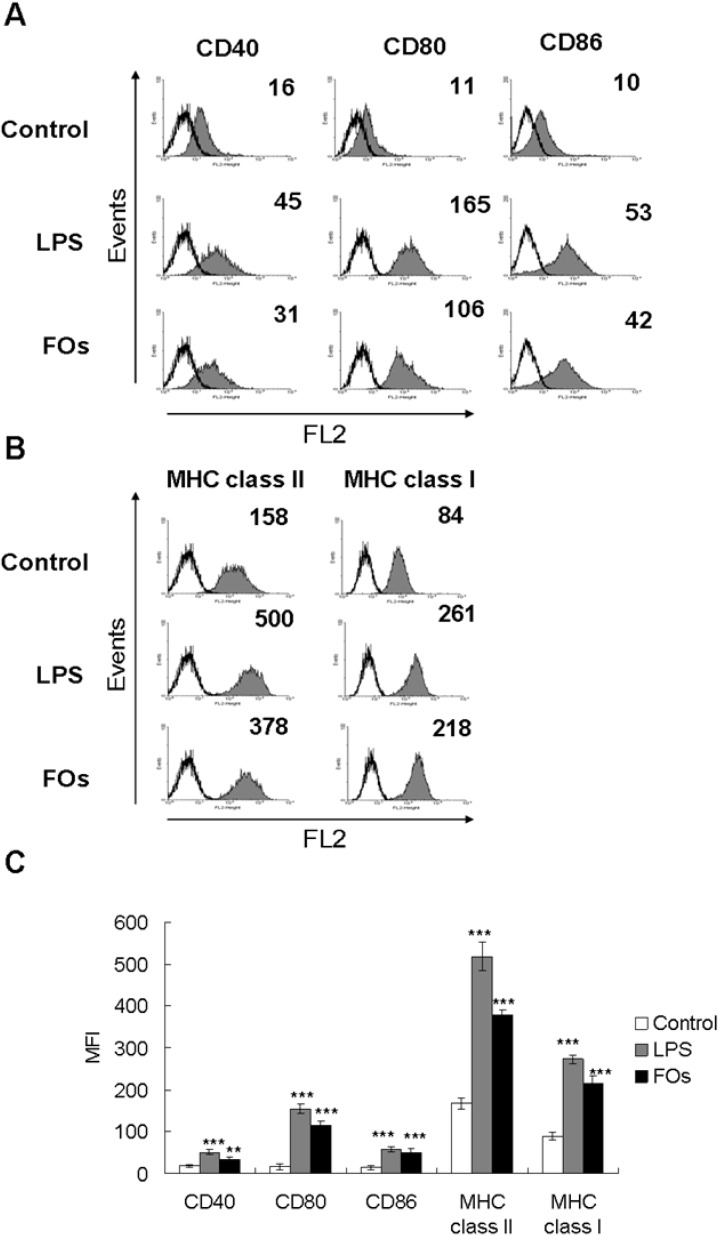
FOs up-regulate the expression of co-stimulatory molecules and MHC proteins on BMDCs Immature DCs were treated with 50 μg FOs or 100 ng/mL LPS for 24 h. After incubation, cells were stained with specific primary antibodies against (**A**) Co-stimulatory molecules CD40, CD80, CD86 or (**B**) MHC proteins, MHC class I, MHC class II which was followed analyzed by flow cytometry. The gray-filled area represents staining with specific primary antibody. The open histogram represents staining with isotype control group. Data were shown as the mean fluorescence intensity (MFI). The histogram shows data from one representative experiment of each group. (**C**) The bar graphs represent the mean ± SD from triplicate well measurements from one of three independent experiments with similar results. ** *p* < 0.01, *** *p* < 0.001; *versus* PBS-treated DC group.

The maturation status of BMDCs was also indicated by the enhanced expression of surface molecules on CD11c^+^ cells. As shown in Figure 2, FOs (50 μg/mL) stimulation of BMDCs resulted in significant up-regulation of co-stimulatory molecules (CD80, CD86 and CD40) and major histocompatibility complex molecules (MHC class II and MHC class I) within 24 h compared to the control group. The increased MHC class I and MHC class II and CD80/86/40 expression levels provided further evidence that FOs promote DC maturation. Taken together, FOs induces phenotypic and functional maturation of DCs as evident from an increase in the expression of co-stimulatory molecules and MHC, and stimulation of cytokines and chemokines.

### 2.3. FOs Increase the Ability of BMDCs to Stimulate OVA-Specific T-cell Proliferation

**Figure 3:**
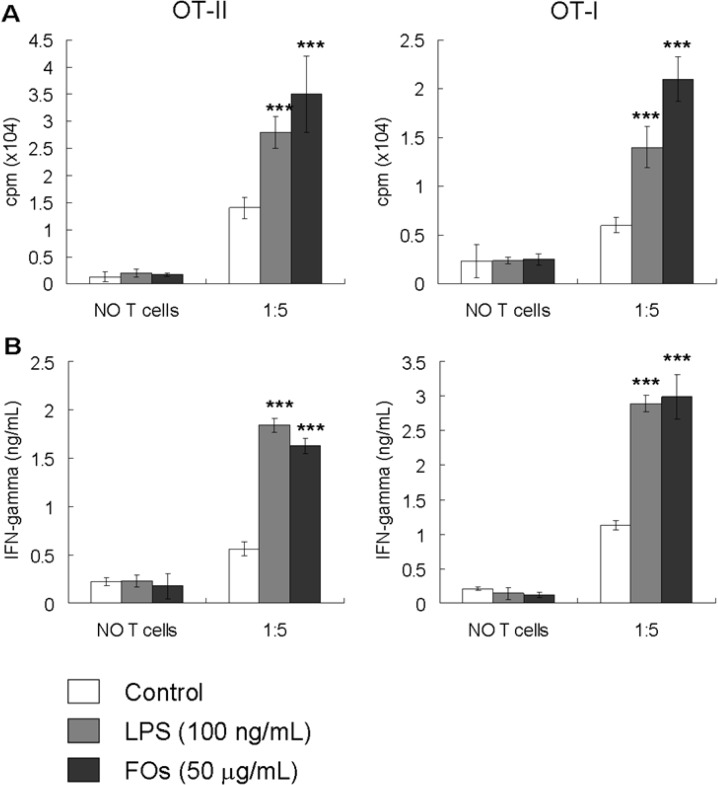
FOs-treated BMDCs increase T cell activation in response to the specific antigen OVA (**A**) CD8^+^ T cells and CD4^+^ T cells were prepared from the spleens of OT-I and OT-II mice, respectively. Purified T cells were co-cultured with PBS, LPS (100 ng/mL) or 50 g/mL FOs-treated DCs pulsed with an OVA peptide at the indicated ratio of DCs: T cells for 96 h. Cell proliferation was quantified by [^3^H]-thymidine incorporation for 18 h. (**B**) Supernatants were collected from cultures after 96 h, and IFN-γ production was measured by ELISA. All data represent the mean ± SD from triplicate well measurements from one of three independent experiments with similar results. *** *p* < 0.001; *versus* PBS-treated control groups.

It has been reported that fully mature DCs induce higher levels of allogenic T cell proliferation as compared to immature DCs [11]. To further compare the stimulatory properties of DCs cultured with FOs, the effect of FOs on BMDCs-mediated activation of OVA antigen-specific T cell responses was examined. FOs-treated, OVA_257–264_ (OVAP_1_) or OVA_323–339_ (OVAP_2_) peptide-loaded BMDCs were co-cultured with their syngeneic OVA-specific CD4^+^ OT-II or CD8^+^ OT-I T cells, and T cell proliferation was measured by [^3^H] thymidine incorporation. The results showed that FOs-activated BMDCs effectively induced enhanced OVA-specific CD4^+^ (OT-II) and CD8^+^ (OT-I) T proliferative responses *in vitro* (Figure 3A). Therefore, the level of IFN-γ in the culture supernatant was also measured using ELISA. As shown in Figure 3B, FOs treatment also increased the amount of IFN-γ produced by the activated CD4^+^ and CD8^+^ T cells. These results revealed that FOs enhance the ability of DCs to induce Ag-specific T cell immune responses.

### 2.4. FOs is Involved with ERK, p38, JNK Kinases, and NF-κB p65 Activation

**Figure 4:**
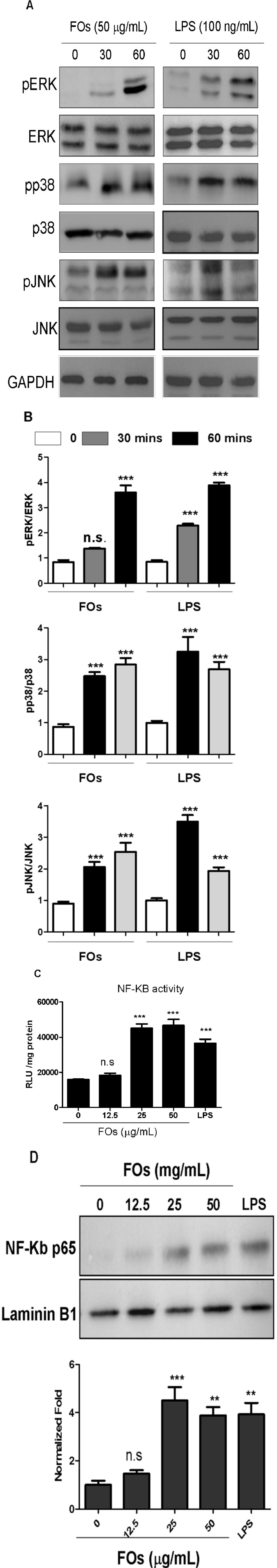
FOs induces MAPK phsophorylation and NF-κB activation in BMDCs. (**A**) Immature DCs were treated with 50 g FOs or 100 ng/LPS and whole cell lystes fractions were collected at indicated time points. Western blotting was performed to determine the phosphorylated or non-phosphorylated ERK, JNK, and p38 using phosphor specific *Abs*, respectively. (**B**) pERK, pp38, pJNK levels were normalized with total ERK, p38 and JNK levels and then compared with the PBS treated control group arranged as one unit. (**C**) 3 h after LPS or FOs treatment, cell lysates were prepared and luciferase activity was measured. Data represent NF-κB-luciferase activity normalized per unit (relative light unit per second) of extract protein. (**D**) NF-κB p65 in nuclear extracts as mentioned was performed as described in material and methods. p65 levels were normalized with laminin B1 and then compared with the PBS treated control group arranged as one unit.

MAPKs (ERK, p38 and JNK) and NF-κB are known to be associated with proinflammatory cytokine gene expression and play a crucial role in DCs maturation [12,13]. To investigate the molecular mechanism underlying the effects of FOs, we sought to study the effect of FOs treatment on the MAPKs and NF-κB pathways in BMDCs. BMDCs were stimulated with FOs, and western blotting analysis was performed to detect the levels of MAPK-ERK, p38, JNK phosphorylation and NF-κB luciferase reporter gene expression and nuclear translocation. As shown in Figure 4A, both FOs and LPS can up-regulate the phosphorylation of the MAPKs ERK, p38 and JNK between 30 and 60 min after treatment, whereas the levels of the nonphosphorylated proteins were not affected. To further determine whether FOs increase the activation of NF-κB, the results showed that FOs and LPS dose-dependently increase NF-κB–dependent luciferase enzyme expression (Figure 4B) and NF-κB p65 nuclear translocation (Figure 4C). Since LPS stimulation of TLR-4 signaling activates MAPKs and NF-κB signal pathways has been well known, total extracts or nuclear extracts from cells prepared with LPS treatment to serve as a positive control.

The graphs shown represent the mean ± SD from three independent experiments with similar results. ** *p* < 0.01, *** *p* < 0.001; *versus* PBS treated control groups.

**Figure 5:**
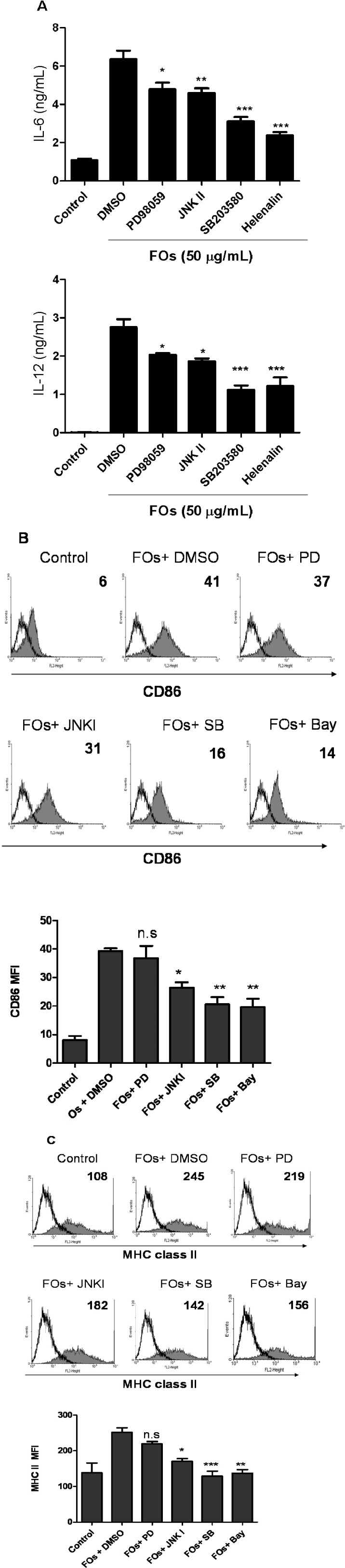
FOs-induced DC maturation was inhibited by PD98059, SB203580, JNK I, BAY 11-7082. Immature DCs were pre-treated with PD98058 (10 μM), JNK I (10 μM), SB203580 (10 μM), and BAY 11-7082 (10 μM) for 1 h before FOs treatment. (**A**) IL-12 cytokine and IL-6 production and (**B**) CD86 and (**C**) MHC class II expression were measured 24 h later.

Next, to further study the roles of these signaling pathway in FOs-induced DCs maturation, we treated DCs with chemical inhibitors against MAPKs and NF-κB. Immature BMDCs were pretreated with SB203580 (a specific blocker of p38 MAPK), PD98059 (an inhibitor of the ERK pathway), JNK I (an inhibitor of the JNK pathway), or BAY 11-7082 (a specific blocker of NF-κB) for 1h and subsequently stimulated with FOs (50 μg/mL) for 24 h. As shown in Figure 5, IL-6 and IL-12 secretion (Figure 5A), CD86 (Figure 5B), and MHC class II (Figure 5C) expression induced by FOs was significantly decreased by SB203580, JNK I and BAY 11-7082. In addition, PD98059 can decreased IL-6 and IL-12 cytokine production, but did not affect the CD86 and MHC class II expression. Therefore, these results suggest that FOs induce DCs activation, possibly through activation of the p38 and JNK MAPK family members and NF-κB pathways, which potentially explains the activating effect of FOs on DC maturation.

The gray-filled area represents staining with specific primary antibody. The open histogram represents staining with isotype control group. Data were shown as the mean fluorescence intensity (MFI). The histogram shows data from one representative experiment of each group. The bar graphs represent the mean ± SD from triplicate well measurements from one of three independent experiments with similar results. * *p* < 0.05, ** *p* < 0.01, *** *p* < 0.001 *versus* DMSO+ FOs treated groups.

### 2.5. BMDCs from TLR 4/2-Deficient Mice do not Respond to FOs Stimulation

**Figure 6:**
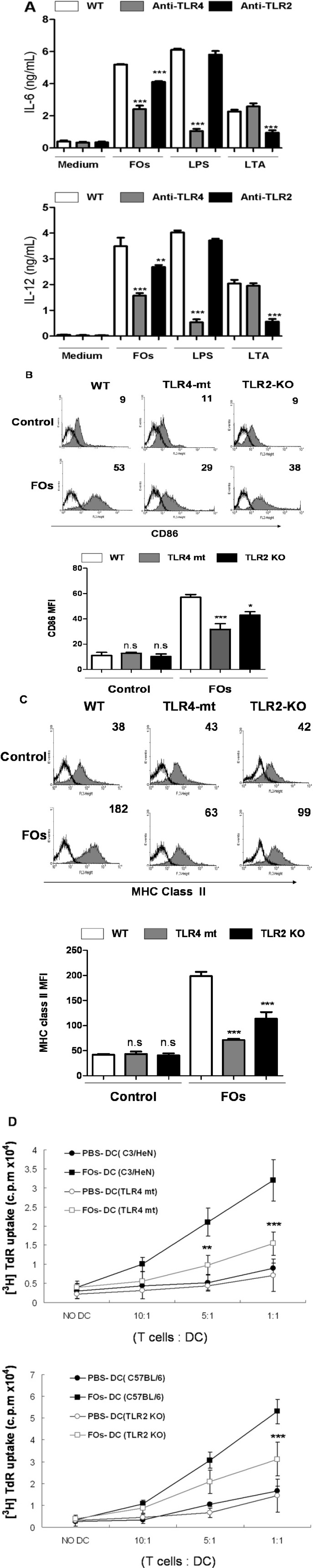
FOs induces IL-6, IL-12 expression and CD86 and MHC class II expression through a TLR-4 and/or TLR-2-dependent signaling pathway. BMDCs were harvested from C3H/HeN, C3H/HeJ (TLR-4-deficient), C57BL/6, or TLR-2 KO mice and stimulated with FOs (50 μg/mL), LPS (100 ng/mL) or LTA (1 μg/mL). (**A**) IL-12 cytokine and IL-6 production and (**B**) CD86 (**C**) MHC class II expression were measured 24 h later. The gray-filled area represents staining with specific primary antibody. The open histogram represents staining with isotype control group. Data were shown as the mean fluorescence intensity (MFI). The histogram shows data from one representative experiment of each group. (**C**) The bar graphs represent the mean ± SD from triplicate well measurements from one of three independent experiments with similar results. (**D**) FOs induces the capability of stimulating allogeneic T-cell response in MLR of BMDCs. T cells were prepared from the spleens of naïve C3/HeN or C57BL/6 mice. Purified T cells were then cocultured with FOs (50 μg/mL), LPS (100 ng/mL) or LTA (1 μg/mL) treated TLR wild type or mutant BMDCs at the indicated ratio of DCs: T cells for 96 h. Cell proliferation was measured by [^3^H]-thymidine incorporation for 18 h. * *p* < 0.05 * *p* < 0.05, ** *p* < 0.01, *** *p* < 0.001 for the comparison between stimulated BMDCs from the mutant mice and their relevant wild type control group.

Several saccharide components were reported to induce maturation ofDCs via the TLR-2 and TLR-4 [14,15,16]. The TLR-4-deficient (C3H/HeJ) and TLR-2 −/− knockout (C57BL/6 background) mouse strains are nonresponsive to LPS and lipoteichoic acid (LTA) due to a point mutation or knockout of their TLR-4 or TLR-2 receptor genes, respectively. These mice were used to further characterize the function of TLR-4 or TLR-2 in FOs-mediated DCs stimulation. Bone marrow-derived DCs from C3H/HeJ or TLR-2 −/− mice and wild-type C3H/HeN or C57BL/6 mice were prepared and phenotypically analyzed. As shown in Figure 6, FOs stimulated IL-6 and IL-12 production (Figure 6A), CD86 (Figure 6B) and MHC class II expression (Figure 6C) and allogeneic T cellsproliferation (Figure 6D) in wild-type DCs, however, these maturation markers was dramatically reduced in BMDCs from both TLR-4-deficient and TLR-2 KO mice. These results show that FOs can induce BMDCs activation at least through interaction with TLR-4 and/or TLR-2 molecules.

## 3. Discussion

In our previous study, we demonstrated the immunostimulatory effect of FOs on macrophages, by showing that FOs induce the production of proinflammatory mediators in RAW264.7 [4]. We found that FOs (0.1–100 μg/mL) induced TNF-α, IL-1β, IL-6, nitric oxide and PGE2 production in unstimulated RAW 264.7 in a dose-dependent manner. However, no investigation has clarified the molecular mechanisms responsible for the regulation of DCs in their activation and maturation states by FOs. DCs are a crucial cell type that acts at the interface of innate and adaptive immune responses and has the unique ability to activate naïve T cells [5,6,7]. Potent modulation of the activation and function of this essential cell type might have potential efficacy against tumor or virus infection or represent a candidate-adjuvant approach for application in immunotherapy and vaccination [9]. In this study, we confirmed the stimulating effect of FOs isolated from rice bran on DCs. Some phenotypic and functional changes were observed to occur in FOs-treated DCs: (1) increased cytokine and chemokine production; (2) increased expressions of MHC-I/II and co-stimulatory molecules (CD40 and CD 80/86); and (3) an increased capacity to induce T cell proliferation.

In immune responses, IL-12 plays a central role as a link between the innate and adaptive immune systems [17]. This cytokine can polarize the immune system toward a primary T helper cell type 1 (Th1) response. In this study, we found that FOs can induce IL-12 production in BMDCs (Figure 1). In addition, co-culture of FOs –stimulated OVA peptide-pulse BMDCs and OVA-specific T cells can significantly increase IFN-γ production (Figure 3), which is a major product of Th1 cells [18]. Therefore, these results indicate that FOs-stimulated DCs preferentially promote Th1 immune responses. On the other hand, because the Th 1 cells producing IFN-γ have been shown to exert a powerful antitumor effect, thus further preclinical studies testing the antitumor effect of FOs is also needed to confirm the *in vivo* study results.

Several known chemokines induce the migration of DCs and may therefore contribute to their recruitment during peripheral immune responses [19]. Our results showed that FOs also can enhance chemokine production in DCs and could thereby increase the chemoattraction of immune cells around DCs, thus creating an ideal microenvironment for activation of adaptive immunity.

Our present study showed that FOs induce NF-κB signaling pathways, and result in DCs maturation (Figure 4). Because NF-κB binding sites are found in the promoter regions of various proinflammatory cytokines, including TNF-alpha, IL-6 and IL-12 [20,21,22], and of co-stimulatory molecules, such as CD40 and CD86 [23], indicating that the NF-κB pathway may be involved in FOs-induced DCs activation. In addition, our present study also showed that FOs can increase ERK, p38 and JNK MPAK phosphorylation. However, in the specific inhibitor study, SB203580 and JNKI, but not PD98059, decreased the CD86 and MHC class II expression by FOs-stimulated DCs (Figure 2C). It has been showed that LPS can upregulated all three MAPKs phosphorylation in DCs; however, inhibition of ERK1/2 by PD98059 does not affect DCs activation but rather regulates DCs survival. In contrast, inhibition of p38 by SB203580 strongly reduces the LPS-induced DCs maturation [24,25]. Thus, we suggest that p38 and JNK pathways could be involved in the active effect of FOs-induced DCs maturation in this study. However, we cannot exclude the dose of these inhibitor incompletely inhibit the phosphorylation of MAPK and NF-κB activity in FOs treated BMDCs. Therefore, in order to further confirm the involvement of MAPK or NF-κB pathway by other tools, such as small interfering RNAs (siRNAs) or short hairpin RNAs (shRNAs) technology is required.

As described before, the stimulation of TLRs leads to the activation of NF-κB and several MAPK pathways, suggesting that TLRs have or share a common signaling pathway [24,26]. The activation of a MAPK pathway subsequently induces gene expression by activating several transcription factors, including NF-κB [27]. In addition, some complex carbohydrates, such as LPS and peptidoglycan, were recognized by TLR-2 or TLR-4 [28,29]. Therefore, our results suggest that FOs induced DCs maturation at least in part via TLR-2 and TLR-4 and resulting in the activation of NF-κB and ERK and JNK MAPK pathways, however, all of these possibilities require further study.

We identified FOs as potential ligands for TLR-4 and TLR-2. Because TLR-4 is one of the major components of the receptor for LPS to promote DCs activation [30], the possibility of contamination with the endotoxin LPS in the FOs preparations used in this study required examination. LAL assays showed an endotoxin content of <0.1 ng/mL in our 1 mg/mL FOs stock solutions, resulting in a maximum possible contamination throughout the assays of 0.01 ng/mL in the 100 μg/mL concentration of FOs. Our previous study showed that DCs failed to mature in response to LPS concentrations of <0.1 ng/mL [31]. Furthermore, in blocking experiments for LPS, BMDCs were incubated with 10 μg/mL of the LPS inhibitor polymyxin B (PMB) prior to FOs stimulation. PMB did not significantly affect the FOs-induced IL-6 and IL-12 production but significantly inhibited the LPS effect in the same experiment (Supplemental Figure 1). Collectively, these data suggest that binding of FOs to TLR-4 and/or TLR-2 could effectively induce the significant activation and maturation of DCs.

## 4. Experimental

### 4.1. Mice and Cell Cultures

Five to eight-week-old specific pathogen-free female C57BL/6, C3H/HeN and C3H/HeJ (TLR-4 mutant) mice were purchased from the National Laboratory Animal Center (Taipei, Taiwan). TLR-2 knockout mice were provided by Dr. Chih-Peng Chang (NCKU, Tainan, Taiwan). OT-I TCR transgenic mice were purchased from the Jackson Laboratory (Bar Harbor, ME, USA). OT-II TCR transgenic mice were provided by Dr. Clifford Lowell (UCSF, San Francisco, CA, USA). NF-κB/luciferase transgenic mice which carry the luciferase gene under the control of NF-κB were provided by Dr. Chuan-Mu Chen. All mice were housed in the barrier facility at Taichung Veterans General Hospital (Taichung, Taiwan) in accordance with the Institutional Animal Care and Use Committee guidelines for animal experimentation, and all procedures were performed in accordance with the Institutional Animal Care and Use Committee guidelines for animal experimentation. Mouse DCs were generated from bone marrow as previously described [32]. Bone marrow (BM) cells were flushed from the femurs and tibias of mice and pooled from two to three mice per strain for each experiment. Red blood cells were lyzed with ammonium chloride, and then washed with PBS. BM cells were suspended in RPMI-1640 medium supplemented with 10% heat-inactivated fetal bovine serum, 100 U/mL penicillin G, 100 μg/mL streptomycin, 2 mM L-glutamine, 20 ng/mL recombinant mouse granulocyte-monocyte colony-stimulating factor (Peprotech, Rocky Hill, NJ, USA) and 20 ng/mL recombinant mouse IL-4 (Peprotech) and cultured in 24-well plates (5 × 10^5^ cells/mL). Fresh medium was supplied every 2 days and non-adherent cells were harvested on day 7 as immature DCs.

### 4.2. Feruloylated Oligosaccharides

FOs are obtained from rice bran treated with HCl hydrolysis, according to the previous method with modification [33]. Weighed rice bran (1 g) was treated with 20 mL 200 mM HCl at 100 °C for 1 h. After centrifugation, the supernatant was filtered and applied to an open column packed with Amberlite XAD-2 (previously washed with 95% *v/v* ethanol and then water). Elution was successively carried out with two column volumes of distilled water, three column volumes of 50% *v/v* methanol and two column volumes of methanol. The fraction eluted by methanol/water was concentrated and lyophilized to get the dry FOs for further analysis. The yield was 0.0358g/g rice bran. The acryl ferulic group was released by mild alkaline treatment of FOs. FOs (100 μL, 1 mg/mL) were hydrolysed with NaOH (100 μL, 0.4 mol/L) for 2 h in the dark at room temperature. The reaction was stopped by adding H_3_PO_4_ (150 μL, 0.4 mol/L). This solution was analysed for ferulic acid by HPLC (Shimadzu LC-10AD, Kyoto, Japan) using a C-18 column (5 μm, 4.6 mm × 250 mm, Mightysil RP-18 GP, Kanto Chemicals, Tokyo, Japan). The column was maintained at 30 °C. A sample volume of 10 μL was injected into the column and eluted with methanol/water/acetic acid (50:50:0.5, *v/v/v*) at a flow rate of 0.8 mL/min for 15 min. The absorbance of the eluate was monitored continuously at 320 nm. Ferulic acid released from FOs was identified by comparison of its relative retention time with a ferulic acid standard. The result showed that the quantity of the acyl ferulic group in FOs was about 15.31 mg/g FO (~548 μg/g of rice bran). To remove endotoxins (lipopolysaccharides or LPS), the sample preparations were passed through an EndoTrap Blue column (Hyglos, Bernried, Germany). The endotoxin activity in the sample preparation was determined by a quantitative, chromogenic QCL-1000 Limulus amoebocyte lysate (LAL) assay (Cambrex Bio Science Walkersville, Inc., Walkersville, MD, USA) according to the manufacturer’s protocol and was found to be <0.1 ng/mg FOs.

### 4.3. In Vitro dcs Activation

Immature DCs were cultured at a density of 1 × 10^6^ cells/well in 24-well plates in medium alone or in the presence of 100 ng/mL LPS (*Escherichia coli*, serotype O26:B6, Sigma St. Louis, MO, USA), 1 μg/mL lipoteichoic acid (LTA; L2515, from *Staphylococcus aureus*, Sigma) or FOs (6.25–100 μg/mL) for 24 h (or 6 h for the TNF-alpha ELISA). The cells were then used for further immunophenotyping, the cytokine production assay or the addition of T cells for DCs-T co-culture experiments. 

### 4.4. Flow Cytometric Analysis of Surface Markers

After stimulation, DCs were harvested and stained with FITC-conjugated mouse CD11c^+^ (clone N418) or phycoerythrin (PE)-conjugated anti-mouse CD40 (clone 3/23), anti-mouse CD80 (clone 16-10A1), anti-mouse CD86 (clone GL1), anti-mouse MHC class I (clone AF6-88.5), anti-mouse MHC class II (clone M5/114.15.2) or isotype-matched control mAbs (clone MOPC-173), all from Biolegend, San Diego, CA, USA, for 45 min on ice (1 μg/mL diluted in PBS/1.0% FCS(*v/v*)). After washing with PBS, the fluorescence intensity was measured with a FACSCalibur flow cytometer (BD Biosciences, Heidelberg, Germany), and the data were analyzed using WINMDI software (Scripps, La Jolla, CA, USA). The results are expressed in terms of the relative mean fluorescence intensity (MFI) on CD11c^+^ gated DCs.

### 4.5. Cytokine Detection

To quantify the production of cytokines and chemokines, supernatants were collected from immature DCs propagated in the presence ofLPS or FOs. After incubation, cytokines (mTNF-alpha, mIL-6, and mIL-12, mIL-10) production and chemokines (MIP-1 alpha and RANTES) by DCs were measured in the supernatants by sandwich ELISA assays according to the manufacturer’s specifications (all from PeproTech, Rocky Hill, NJ, USA).

### 4.6. Neutralization Experiments

BMDCs were preincubated with 10 μM SB203580 (a specific blocker of p38 MAPK, Sigma-Aldrich, St Louis, MO, USA), 10 μM PD98059 (an inhibitor of the ERK pathway, Sigma-Aldrich), 10 μM JNK I (an inhibitor of the JNK pathway, Calbiochem, San Diego, CA,USA), or 10 μM BAY 11-7082 (a specific blocker of NF-κB, sigma) for 1 h followed by FOs (50 μg/mL) treatments for 24 h. The conditioned media were collected and analyzed for IL-6 and IL-12 secretion by ELISA. The cells were harvested and analyzed surface marker CD86 and MHC class II expression by flow cytometry. The doses of these inhibitors are referred to previous reports [25,34]. Furthermore, in blocking experiments for LPS were also according to previous reports [35,36]. In brief, BMDCs were incubated with 10 μg/mL of the LPS inhibitor polymyxin B (PMB) prior to FOs stimulation.

### 4.7. OVA-Specific T Cell Activation

Immature DCs from C57BL/6 mice were pulsed with 2 μg/mL OVA_257–264_ (OVAP_1_) or OVA_323–339_ (OVAP_2_) (synthesized by Echo Chemical Co., Miaoli, Taiwan) in the presence of LPS or FOs (50 μg/mL) for 24 h. FOs were then washed out, and OVAP_1_-specific CD8^+^ T cells(2 × 10^5^) and OVAP_2_—specific CD4^+^ T cells (2 × 10^5^) were added to the culture at 1:5 DCs:T cell ratio in 96-well round-bottom plates. The OVA-specific CD4^+^ and CD8^+^ T cells were positively enriched from the pooled spleens of two to three OT-1 and OT-2 mice by EasySep^®^ Mouse CD8a Positive Selection Kit separation and EasySep^®^ Mouse CD4 Positive Selection Kit separation, respectively, according to the manufacturer’s protocol (Stem Cell Technologies, Grenoble, France). After an additional 96 h of co-incubation, T cell proliferation was measured by adding 1 μCi of [^3^H] thymidine (GE Healthcare, Buckinghamshire, UK) for overnight incubation; quantification of incorporated [^3^H] thymidine was subsequently performed by liquid scintillation counting on a β-Counter (Beckman Instruments, Palo Alto, CA, USA) [32]. In addition, the IFN-γ ELISA kit (eBioscience, San Diego, CA, USA) was also used following the manufacturer’s protocol.

### 4.8. Allogenic Mixed Lymphocyte Reaction

Mouse T cells were isolated from naïve C57BL/6, C3/HeNmice spleens using EasySep Mouse T Cell Enrichment Kit separation, respectively, according to the manufacturer’s protocol (Stem Cell Technologies). Immature BMDCs were generated from C57BL/6, C3H/HeN, C3H/HeJ and TLR-2 KO mice and then stimulated with LPS (100 ng/mL), LTA (1 μg/mL) or FOs (50 μg/mL) for 24 h. After stimulation the cells were harvested, washed, and diluted with the prepared enriched T cells (2 × 10^5^) in ratios of 10:1, 5:1 and 1:1 (T cells: DC) in 96-well round-bottom plates (Corning, NY, USA). After an additional 96 h of co-incubation, T cell proliferation was measured by adding 1 μCi of [^3^H] thymidine (GE Healthcare) for overnight incubation; quantification of incorporated [^3^H] thymidine was subsequently performed by liquid scintillation counting on a β-Counter (Beckman Instruments, Palo Alto, CA, USA).

### 4.9. Activity Assay for MAPKs and NF-κB

Immature DCs were stimulated with 50 μg/mL FOs, and whole cell lysates and nuclearfraction extracts were prepared at the indicated time points as previously described [33]. The protease inhibitors leupeptin (Sigma-Aldrich) and aprotinin (Sigma-Aldrich) were used at a concentration of 10 μg/mL in all steps. Protein concentrations were determined using a BCA protein assay kit (Pierce, Rockford, IL, USA) prior to western blot or other protein analysis. For protein detection, protein extracts (40 g/mL) were boiled separated on 10% SDS-polyacrylamide gels and electrotransferred to nitrocellulose membranes. The membranes were blocked for 1 h with 5% skim milk in TBS + 0.05% Tween 20. After blocking, the membranes were incubated overnight with the suggested concentrations of either a primary antibody against phospho-p38, p38, phospho-p42/44, total p42/44, phospho-JNK, NF-κB p65, Lamin B1, β-Actin (all purchased from Cell Signaling Technology, Beverly, MA, USA). The membranes were washed prior to incubation with HRP-Conjugated Goat Anti-Mouse IgG (115-035-068) or HRP-Conjugated Goat Anti-Rabbit IgG secondary Abs (111-035-003) (all purchased from Jackson ImmunoResearch, West Grove, PA, USA). The proteins were detected by enhanced chemiluminescence (GE Healthcare) and analyzed using the LAS3000 system (Fujifilm, Tokyo, Japan). Densitometric analysis was performed with ImageJ software (National Institute of Health, Bethesda, MD, USA). Active forms of MAPKs (pERK, p38 and pJNK) were normalized with corresponding total nonphosphorylated forms. To examine the *NF-κB* transcriptional *activity*, the Bone marrow derived DCs were generated from NF-κB-luciferase transgenic mice as above described. Cell were incubated with 100 ng/mL LPS or 50 μg/mLFOs for 3 h. Cell extracts were prepared using luciferase cell lysis buffer (Promega, Madison, WI, USA ) and mix 20 μL cell extracts with 100 μL of luciferase assay reagent (Promega). Then, the lucifease activity was counted for 10 s in a Turner BioSystems luminometer (Promega, Sunnyvale, CA, USA) and results were normalized per unit (relative light unit per second) of extract protein.

### 4.10. Statistical Analysis

All statistical analyses were performed using the GraphPad Prism software package version 4.0. The statistical analyses of cytokine production, surface marker expression, T cell proliferation and western blotting and luciferase assay were performed using one-way ANOVA followed by Tukey’s post-hoc test. A *p* < 0.05 was considered statistically significant.

## 5. Conclusions

In summary, for the first time, we present evidence demonstrating that FOs of rice bran can augment DCs maturation in an *in vitro* culture system. However, we also believe that additional issues warrant future investigation. In particular, the details of the molecular mechanisms underlying the ability of FOs to induce DCs function are poorly understood that remains to be determined. In addition, although our results showed that FOs enhances DCs function *in vitro*, it remains unknown how this is effective under in oral administration in animals.

## Abbreviations

FOs: Feruloylated oligosaccharides
DCs: dendritic cells
MAPK: mitogen-activated protein kinase
TLR: Toll-like receptor
FA: ferulic acid
APCs: antigen-presenting cells
MHC: major histocompatibility complex
BMDCs: bone marrow-derived dendritic cells
ERK: extracellular signal-regulated kinase
JNK: c-JUN N-terminal kinase
NF-κB: nuclear factor kappa-light-chain-enhancer of activated B cells
TNF-alpha: tumor necrosis factor
IL: interleukin
TH: T helper cells
IFN-γ: interferon-γ
LPS: lipopolysaccharides
LTA: lipoteichoic acid
MFI: mean fluorescence intensity
OVA: ovalbumin
MCP-1: monocyte chemotactic protein-1
MIP-1β: macrophage inflammatory protein-1 beta
RANTES: regulated on activation; normal T cell expressed and secreted

## Supplementary Materials

Supplementary materials can be accessed at: http://www.mdpi.com/1420-3049/19/4/5325/s1.
