# Supplementary material for: Rice Bran Feruloylated Oligosaccharides Activate Dendritic Cells via Toll-Like Receptor 2 and 4 Signaling
Source: Molecules. 2014 Apr 23;19(4):5325–47. doi: 10.3390/molecules19045325 (PMC6271473; doi:10.3390/molecules19045325)

Supplemental Materials

Figure S1. The stimulatory activity of FOs on BMDCs is not due to contamination. Immature BMDCs, which were pre-incubated alone or in the presence of PmB (100 μg/mL) for 2 h at 37 ℃, were stimulated with Fos (50 g/mL) or LPS (100 ng/mL) for 24 h. (**A**) Supernatants were collected, and mouse Il-6 and IL-12 production were measured by ELISA. (**B**) The expression of the surface marker CD80 was analyzed by flow cytometry with a fluorescently labeled Ab. The gray-filled area represents staining with specific primary antibody. The red-line represents PMB treated group. The open histogram represents staining with isotype control group. Data were shown as the mean fluorescence intensity (MFI). The histogram shows data from one representative experiment of each group. (**C**) The bar graphs represent the mean ± SD from triplicate well measurements from one of three independent experiments with similar results. *** $p < 0.001$; *versus* FOs or LPS treated alone DCs group.

A

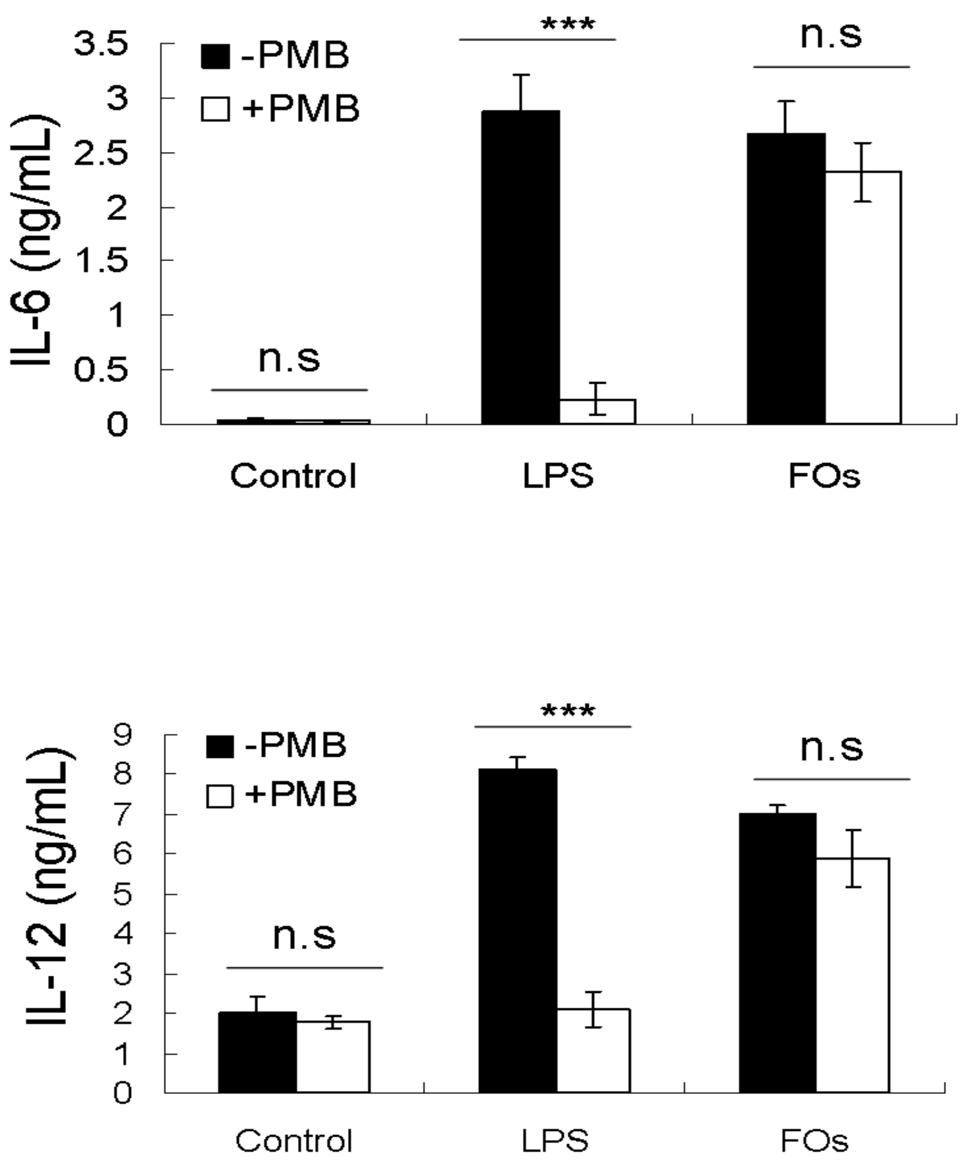

Figure S1. *Cont.*

B

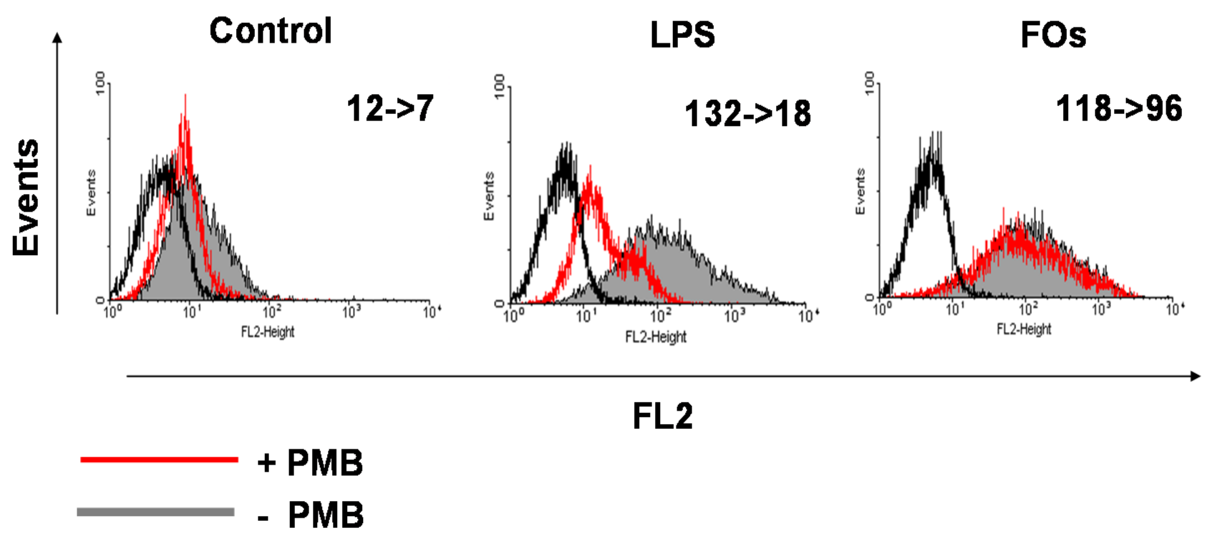

C

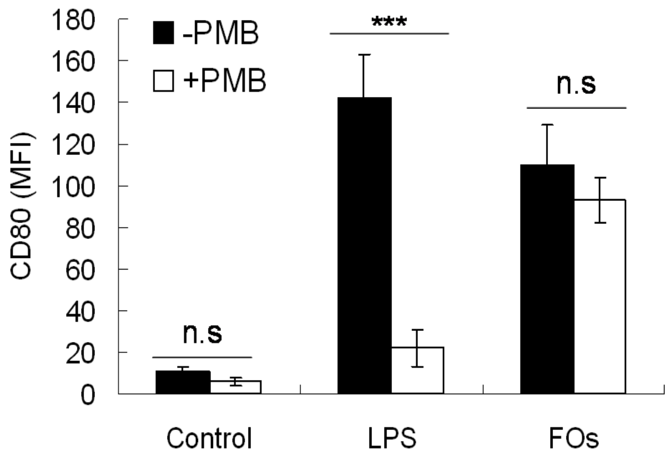

Supplement: Supplementary file 1 [file molecules-19-05325-s001.pdf]
